# Neural Networks Application for the Data of PID Controller for Acrobot

**DOI:** 10.1155/2022/9162352

**Published:** 2022-04-14

**Authors:** Nguyen Cong Danh

**Affiliations:** The Independent Researcher, Pailin, Cambodia

## Abstract

Acrobots are a system that has levels of operating states in many investigated cases, and they are subjects to many events during operation due to the mechanisms of locomotion processes. These states have been investigated in specific situations. Due to the limited nature of surveying under conditions without the aid of software fine-tuning the desired output values, designers have to create a number of algorithms that control the system most appropriately in a complex working environment of this system. In this study, the author has proposed design problems to suit the working needs. The author has modeled the objects to be investigated, and at the same time, the author has combined simulation of phenomena that have been documented from the theoretical model of this system before. Control an Acrobot system, including Neural Network Application for the data of the PID controller (closed form). Mathematical models, Simulink, are also presented specifically through this study. The simulation parameters have been adjusted to match the set criteria, and the reader's perception will be more intuitive through simulation interpretations. Based on simulation data, the system performance analysis becomes more accurate than ever. The above suggestions are intended to serve vocational education and scientific research, and at the same time, they also contribute to suggesting new and unique ideas. The ANN is the most intelligent control method currently added in this study to firmly confirm its effectiveness in all problems related to artificial intelligence. Proposing control strategies for different models is also suggested by the author in the conclusion. Neural networks application is a highly applicable method because it works in sync with other methods. In other words, neural networks application can be applied to any situation based on given data. Specifically, in this study, the application of neural networks becomes more flexible and vivid thanks to the data of the PID controller. The purpose of this method is to increase the security of the system against the attack of hackers on facilities of the automatic control system. Simulation is done by Matlab.

## 1. Introduction

**Figure 1:**
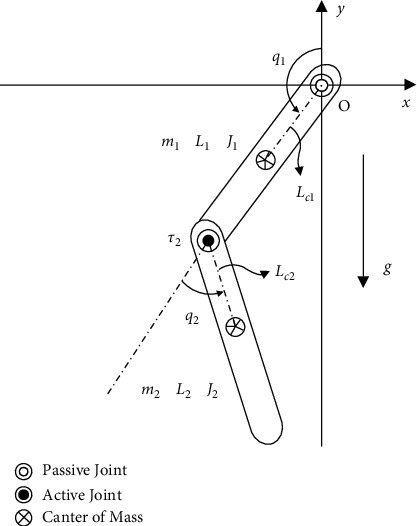
Mathematical model of Acrobot [25].

In the future, in control algorithms, including PID, the artificial intelligence mode can be applied to flexible electric motors, and electromechanical devices have yielded encouraging results. Above control methods are an important component in controlling a flexible Acrobot. Artificial intelligent (Ai) algorithms have also been implemented in labs for Acrobots, and they can appear in industry factories. Flexible robots are controlled by a combination of genetic algorithms [1] and classical algorithms: PID controller, and fuzzy mode to intelligent algorithms: Ai [2]. Broadly understood, Papoutsidakis et al. [2] included algorithms associated with neural networks. These algorithms will be applied to a small branch of robotic feet. Research on these control algorithms brings many practical applications such as self-flying fighter and self-driving aircraft carrier. In this study, the author proposed new several control methods to control a relatively complex system, Acrobot. These control methods are viewed as a direct application to Acrobot. Besides, the application of artificial intelligence has been mentioned in this topic. Acrobot systems as well as other systems that are structurally similar to inverted pendulum, Pendubot [3], and reaction wheel inverted pendulum [4], which are nonlinear systems such as single-input multi-output (SIMO). Although they have a similar structure, they have their own ways of operating according to a particular mechanism. In terms of physical theory, the mathematical basis for forming their physical phenomena is different. They are nonlinear systems. They do not follow a linear phenomenon because their model is complex. They have many interactions between metal rods that are joined together. Therefore, the design of controllers for these systems always faces challenges with many signals at the output. Output signals are fair valid only when positions near the working point are considered in this study. Modern control methods are implemented according to this rule, including neural networks application. This system is described by 2 metal/hard plastic bars that have a joint between 2 metal/hard plastic bars. The first metal/hard plastic bar is denoted “link 1” and the 2^nd^ metal/hard plastic bar is denoted “link 2.” This coupling is regulated by the motor so that the movement of the system is smooth. This joint is a joint with flexible movement with large opening angle between two bars. The top of “link 1” is attached to the passive joint and “link 1” is moved freely around this passive joint. This passive joint is mounted on a fixed support. The Acrobot system is shown by a link between “link 1” and “link 2” (Figure 1).

## 2. Dynamic Equation of the Model System

All models are represented by physical phenomena. Mathematical equations reflect physical properties and phenomena. Operating states of the system are described by these mathematical equations. Through these equations, the author has established a model with the intervention of (Ai). The purpose of this is the author's wish. The author desired that the system achieves stable values under the control of the controllers. In the future, the controllers can be controlled remotely. This is useful for cases where working conditions are harsh for humans. Then, one or more controllers are connected to a system, and they are controlled by the remote control system. After successfully setting up the mathematical equations, the author has processed to calculate values: the value of the response of the PID controller and the value of neural networks application. After finding above values, the author found the control signals in the open system and closed system for the PID controller. Acrobot or an inverted pendulum can be described by mathematical equations. The two-dimensional coordinate system, Ox and Oy, is shown in Figure 1 [5]. The control method for surveys [6–8] can be applied to nonlinear models such as SIMO. Planar dictubot [9] with the artificial intelligence method is an attractive title for readers. Swing-up control [10] for nonlinear models like SIMO is a good idea. Chaotic perturbation [11] can be applied to nonlinear models such as SIMO. Hybrid control [12] is combined with the neural network to form a new research direction. Application of the ant colony algorithm [13] in optimal control of robotic foot systems is an exciting future work. Energy-based control [14] for the robot's feet through their movement is an elaborate research program. Hybrid control [15] with navigation functions for the inverted pendulum is a great mission. The robust control for stabilization of the noninertial system [16] can be applied to an inverted pendulum. A positioning control strategy [17] is intended for nonlinear systems to examine their behavior more closely. Balance control [18] for Pendubot in various forms is of great interest to the author. The inverse linear quadratic method [19] can be used for nonlinear systems. Several new algorithms have been implemented [20, 21] for controlling robots which can replace neural network applications. Approaches in the processing of signals materialized by abstract images [22] have also been studied in recent years. The development from classical algorithms to more modern algorithms [23, 24] has promoted its effectiveness.

In this study, neural network applications are designed after a linearization process is established, as given in Section 3. The linearization of this model makes the survey more convenient in the process of controlling the properties around the working point as described above. This purpose contributes to the stabilization of nonlinear structures. The challenges of the proposed strategy: the working environment of this system is relatively complex. Therefore, it is necessary to stabilize the structure properties before controlling them with any control method.

Physical parameters of Acrobot are given in Table 1. Variables of the mathematical equation are *q*_1_, *q*_2_, and *τ*_2_, where *q*_1_ and *q*_2_ are the output signals and *τ*_2_ is the input signal. Variables are unknown values. These variables will be solved through the establishment of a mathematical equation presented. In the process of designing controllers for these systems, readers can better understand the nature of the relationships between input signals and output signals through voltage signals, the torque of an electric motor. In Figure 1, the *x*-axis of the Cartesian coordinate system was chosen to be the reference level of zero potential energy. Letting *S*_*i*_=[*S*_*i*_^*x*^, *S*_*i*_^*y*^] ∈ *R*^2^ be the absolute position of the COM of the *i*^th^ link gives [5]

(1) $$\begin{array}{c} S_{1}=\left[\begin{array}{c} Oc_{1}\sin\;q_{1}\\ Oc_{1}\cos\;q_{1} \end{array}\right], \end{array}$$

(2) $$\begin{array}{c} S_{2}=\left[\begin{array}{c} O_{1}\sin\;q_{1}+O_{c2}\sin\left(q_{1}+q_{2}\right)\\ O_{1}\cos\;q_{1}+O_{c2}\cos\left(q_{1}+q_{2}\right) \end{array}\right]. \end{array}$$

The kinetic energy is $Y\left(q,\dot{q}\right)$, and the potential energy is *V*(*q*):

(3) $$\begin{array}{c} Y\left(q,\dot{q}\right)=\frac{1}{2}\sum_{i=1}^{2}\left[m_{i}S_{i}^{2}+J_{1}{\dot{q}}_{1}^{2}+J_{2}{\left({\dot{q}}_{1}+{\dot{q}}_{2}\right)}^{2}\right],\\ Y\left(q,\dot{q}\right)=\frac{1}{2}\left[\begin{array}{cc} {\dot{q}}_{1} & {\dot{q}}_{2} \end{array}\right]M\left(q_{2}\right)\left[\begin{array}{c} {\dot{q}}_{1}\\ {\dot{q}}_{2} \end{array}\right], \end{array}$$

where

(4) $$\begin{array}{c} M\left(q_{2}\right)=\left[\begin{array}{cc} a_{1}+a_{2}+2a_{3}\cos\left(q_{2}\right) & a_{2}+a_{3}\cos\left(q_{2}\right)\\ a_{2}+a_{3}\cos\;q_{2} & a_{2} \end{array}\right], \end{array}$$

(5) $$\begin{array}{c} \left\{\begin{array}{c} a_{1}=m_{1}O_{C1}^{1}+m_{2}O_{1}^{2}+J_{1},\\ a_{2}=m_{2}O_{C2}^{2}+J_{2},\\ a_{3}=m_{2}O_{1}O_{C2},\\ a_{4}=\left(m_{1}O_{c1}+m_{2}O_{1}\right)g,\\ a_{5}=m_{2}O_{c2}g. \end{array}\right. \end{array}$$

Considering friction is very small, the author used Lagrangian of Acrobot; then, the dynamic equation of the mechanical system is

(6) $$\begin{array}{c} V\left(q\right)=m_{1}gS_{1}^{y}+m_{2}gS_{2}^{y}=a_{4}\cos\;q_{1}+a_{5}\cos\left(q_{1}+q_{2}\right), \end{array}$$

(7) $$\begin{array}{c} \frac{d}{dt}\left[\frac{\partial O\left(q,\dot{q}\right)}{\partial {\dot{q}}_{i}}\right]-\frac{\partial O\left(q,\dot{q}\right)}{\partial q_{i}}={\dot{\tau }}_{i},\;i=1,2, \end{array}$$

where $O\left(q,\dot{q}\right)=Y\left(q,\dot{q}\right)-V\left(q\right)$ and *τ*_1_=0. Equation (7) is equivalent to

(8) $$\begin{array}{c} M\left(q_{2}\right)\ddot{q}+C\left(q,\dot{q}\right)\dot{q}+G\left(q\right)=\left[\begin{array}{c} 0\\ \tau_{2} \end{array}\right], \end{array}$$

where

(9) $$\begin{array}{c} q={\left[\begin{array}{cc} q_{1} & q_{2} \end{array}\right]}^{T}, \end{array}$$

(10) $$\begin{array}{c} C\left(q,\dot{q}\right)=\left[\begin{array}{cc} -a_{3}{\dot{q}}_{2}\sin\;q_{2} & -a_{3}\left({\dot{q}}_{1}+{\dot{q}}_{2}\right)\sin\;q_{2}\\ a_{3}{\dot{q}}_{1}\sin\;q_{2} & 0 \end{array}\right], \end{array}$$

(11) $$\begin{array}{c} G\left(q\right)=\left[\begin{array}{c} -a_{4}\sin\;q_{1}-a_{5}\sin\left(q_{1}+q_{2}\right)\\ -a_{5}\sin\left(q_{1}+q_{2}\right) \end{array}\right]. \end{array}$$

State space equation is created as

(12) $$\begin{array}{c} x={\left[\begin{array}{cccc} x_{1} & x_{2} & x_{3} & x_{4} \end{array}\right]}^{T}={\left[\begin{array}{cccc} q_{1} & {\dot{q}}_{1} & q_{2} & {\dot{q}}_{2} \end{array}\right]}^{T}. \end{array}$$

Let

(13) $$\begin{array}{c} H\left(q,\dot{q}\right)={\left[\begin{array}{cc} H_{1}\left(q,\dot{q}\right) & H_{2}\left(q,\dot{q}\right) \end{array}\right]}^{T}≔C\left(q,\dot{q}\right)\dot{q}+G\left(q\right). \end{array}$$

Then, the dynamic equation (8) can be described as

(14) $$\begin{array}{c} \dot{x}=f\left(x\right)+g\left(x\right)\tau_{2}. \end{array}$$

where

(15) $$\begin{array}{c} \left\{\begin{array}{c} f\left(x\right)={\left[\begin{array}{cccc} x_{2} & R\left(1,1\right) & x_{4} & R\left(2,1\right) \end{array}\right]}^{T},\\ g\left(x\right)={\left[\begin{array}{cccc} 0 & I\left(1,1\right) & 0 & I\left(2,1\right) \end{array}\right]}^{T}, \end{array}\right. \end{array}$$

where *R*(*i*, *j*) and *I*(*i*, *j*) are the matrices having the *i*^th^ row and *j*^th^ column of *R* and *I*. Therefore, *R* and *I* are determined as

(16) $$\begin{array}{c} \left\{\begin{array}{c} R=-M^{-1}\left(q_{2}\right)\left[\begin{array}{c} H_{1}\left(q,\dot{q}\right)\\ H_{2}\left(q,\dot{q}\right) \end{array}\right],\\ I=-M^{-1}\left(q_{2}\right)\left[\begin{array}{c} 0\\ 1 \end{array}\right]. \end{array}\right. \end{array}$$

Parameters of the model are given in Table 2.

## 3. The Survey of the System

The state variable equations of the system are described as follows:

(17) $$\begin{array}{c} \left\{\begin{array}{c} \dot{x}=Ax+Bu,\\ c=Cx+Du, \end{array}\right. \end{array}$$

where

(18) $$\begin{array}{c} A=\left[\begin{array}{cccccc} \frac{\partial f_{1}}{\partial x_{1}} & \frac{\partial f_{1}}{\partial x_{2}} & \frac{\partial f_{1}}{\partial x_{3}} & \frac{\partial f_{1}}{\partial x_{4}} & \ldots & \frac{\partial f_{1}}{\partial x_{n}}\\ \frac{\partial f_{2}}{\partial x_{1}} & \frac{\partial f_{2}}{\partial x_{2}} & \frac{\partial f_{2}}{\partial x_{3}} & \frac{\partial f_{2}}{\partial x_{4}} & \ldots & \frac{\partial f_{2}}{\partial x_{n}}\\ \ldots & \ldots & \ldots & \ldots & \ldots & \ldots \\ \frac{\partial f_{1}}{\partial x_{1}} & \frac{\partial f_{1}}{\partial x_{2}} & \frac{\partial f_{1}}{\partial x_{3}} & \frac{\partial f_{1}}{\partial x_{4}} & \ldots & \frac{\partial f_{1}}{\partial x_{n}} \end{array}\right],B=\left[\begin{array}{c} \frac{\partial f_{1}}{\partial u}\\ \frac{\partial f_{2}}{\partial u}\\ \ldots \\ \frac{\partial f_{n}}{\partial u} \end{array}\right]. \end{array}$$

With above parameters, matrices *A* and *B* of the state space model, *G*_1_(*s*), *G*_2_(*s*), are calculated:

(19) $$\begin{array}{c} A=\left[\begin{array}{cccc} 0 & 1 & 0 & 0\\ 70.6961 & -19.6783 & 0 & 0\\ 0 & 0 & 0 & 1\\ -94.9903 & 130.9458 & 0 & 0 \end{array}\right],B=\left[\begin{array}{c} 0\\ -185.7355\\ 0\\ 927.3017 \end{array}\right]C=\left[\begin{array}{cccc} 1 & 0 & 0 & 0\\ 0 & 1 & 0 & 0\\ 0 & 0 & 1 & 0\\ 0 & 0 & 0 & 1 \end{array}\right],D=0. \end{array}$$

The transfer function method is a mathematical description of an automated system that makes it easier to investigate the system. The transfer function of the system is formed by converting the mathematical description from the form of an equation of state to the form of a transfer function. This is done using Matlab software commands. The transfer function of the system *G*_1_, *G*_2_, *G*_3_, *G*_4_. *G*_1_, *G*_2_, *G*_3_, *G*_4_ can be examined separately using the PID controller in each specific case: *G*_1_ is connected to *G*_PID_ and *G*_2_ is connected to *G*_PID_ as follows:

(20) $$\begin{array}{c} G_{1}\left(s\right)=\frac{-185.7}{s^{2}+19.68s-70.7},\\ G_{2}\left(s\right)=\frac{927.3s^{2}-6074s-4.791\times 10^{4}}{s^{4}+19.68s^{3}-70.7s^{2}},\\ G_{3}\left(s\right)=\frac{-185.7s}{s^{2}+19.68s-70.7},\\ G_{4}\left(s\right)=\frac{927.3s^{2}-6074s-4.791\times 10^{4}}{s^{3}+19.68s^{2}-70.7s}. \end{array}$$

## 4. Controller Design Using PID Stratery

PID controllers are commonly used to regulate the time domain behavior of many different types of dynamic plants [26]. The transfer function of PID control is given by

(21) $$\begin{array}{c} G_{\text{PID}}=K_{P}+\frac{K_{I}}{s}+K_{D}s=\frac{K_{D}s^{2}+K_{P}s+K_{I}}{s}=\frac{K_{D}\left[s^{2}+K_{P}/K_{D}s+K_{I}/K_{D}\right]}{s}, \end{array}$$

where *K*_*p*_, *K*_*i*_, and *K*_*d*_ are the parameters of the PID controller. These parameters can be adjusted according to according to the requirements given. *G*_PID_ can be directly connected to the system transfer function.

## 5. Model Using the Artificial Neural Network (ANN)

### 5.1. An Artificial Neural Network

An artificial neural network (or neural network for short) can be seen as a simple mathematical model of the human brain. Neural networks consist of neurons (processing units) connected to each other by links. Each link is associated with weight, which characterizes the excitatory or inhibitory properties between neurons.

#### 5.1.1. Input Signal

There are (*m*) input signals, where (*m* − 1), the excitation signal, at the input is (*x*_1_, *x*_2_,…, *x*_*m* − 1_); they are taken from the output of neurons placed before this neuron or they are taken from other input signal sources. These input excitation signals are passed through a set of weights (*w*) that characterizes the degree of association between the front neurons is associated with it. A positive association weight corresponds to a restrained synapse. Particularly, the (*m*^th^) input signal component (*x*_*m*_) is called threshold with the value *x*_*m*_ =  + 1. The (*x*_*m*_) signal is passed through the displacement component (bias) *b*_*i*_: *w*_*m*_=*b*.

#### 5.1.2. Output of Neurons

The output of the neuron is given by the expression: *y* = *a* (net) = *a* (*f*), where a (·) is the symbol of the conversion function. In the conversion function, there is a document also called activation function or transfer function, which is responsible for converting the total weight (*f*) (or net) into an output signal (*y*).

### 5.2. Multilayer Feedforward Neural Network

#### 5.2.1. Multilayer Feedforward Neural Network Architecture

The multilayer feedforward network is a feedforward network with two or more layers of processing of neurons. The layer of neurons connected to the input is called the input layer (usually the input layer does not perform processing operations), the layer of neurons connected to the output is called the output layer, and the layer of neurons that is not directly connected to the input and the output is called the hidden layer. Connections between neurons in layers can be complete or incomplete. The algorithm for training the multilayer feedforward network is a backpropagation algorithm.

#### 5.2.2. Multilayer Feedforward Neural Network on Matlab

**Figure 2:**
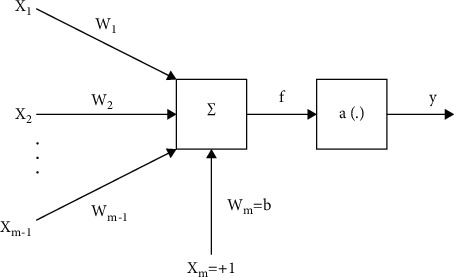
Artificial neurons [27–31].

**Figure 3:**
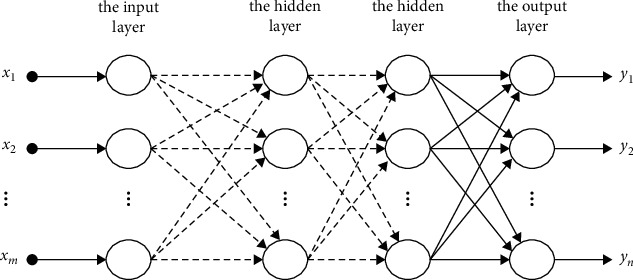
Multilayer feedforward neural network [27–31].

**Figure 4:**
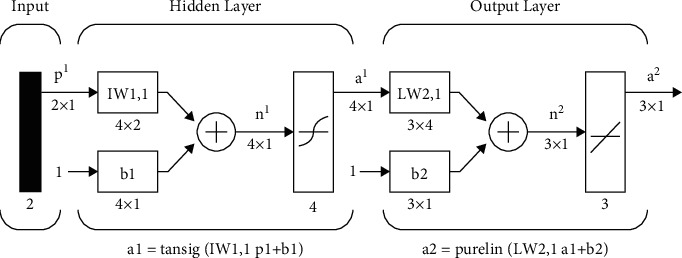
Model of the multilayer feedforward neural network on Simulink [27–31].

To set up the multilayer feedforward neural network below on Matlab, the author used the command “newff,” which takes four input arguments, the first is the value “input” taken from the variable name “in” of “To workspace” in Simulink. This value indicates the range of the input variable, followed by arrays showing the number of neurons per layer: the first layer has fifty neurons, the output layer has one neuron, then the array showing the name of conversion functions used in each layer: the input layer conversion function is “tan-sigmoid,” the output layer is layer linearity, and finally the name of the function used to train the network: the training function is “trainrp,” and parameters of the network are initialized according to the preset algorithm. The command is described as follows: net = newff ([minmax (input)] (Figures 2–4), [50 1],{“tansig” “purelin”},”trainrp”).

## 6. Simulation Results and Discussion

The diagram of the system using the PID controller and simulation results are shown Figures 5–32.

### 6.1. Part 1: Model Using PID Controller

**Figure 6:**
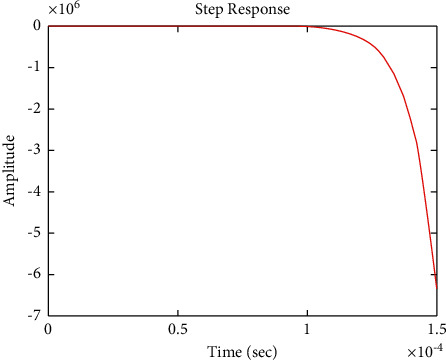
Step response of the closed form for transfer function *G*_1_(*s*).

**Figure 7:**
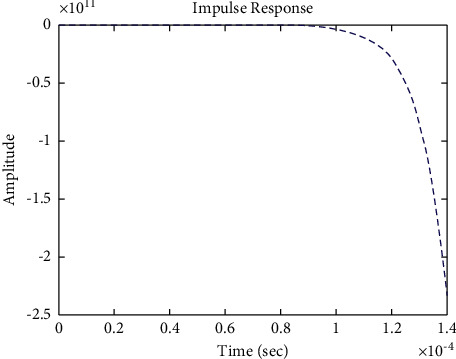
Impulse response of the closed form for transfer function *G*_1_(*s*).

**Figure 8:**
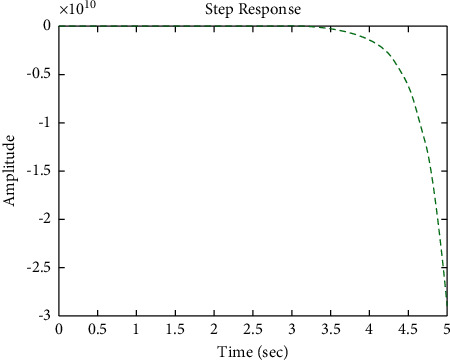
Step response of the open form for transfer function *G*_1_(*s*).

**Figure 9:**
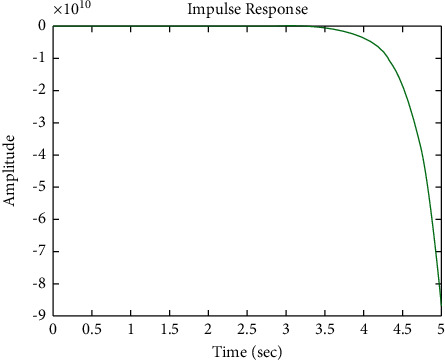
Impulse response of the open form for transfer function *G*_1_(*s*).

Figure 7 shows the impulse response for the closed form (the signal is highlighted in blue) is worse than the open form (the signal is highlighted in green in Figure 9). The value of the amplitude of the closed form in this case is large, and the closed form does not reach a state of the steady. For the PID controller, the closed form does not respond well. The value of the amplitude of the open form in this case is large, and the open form does not reach a state of the steady.  Figure 6 shows the step response for the closed form (the signal is highlighted in red) is better than that for the open form (the signal is highlighted in green in Figure 8). The value of amplitude of the closed form in this case is large, and the closed form does not reach a state of the steady. For the PID controller, the closed form does not respond well. Meanwhile, the open form cannot respond well.

**Figure 11:**
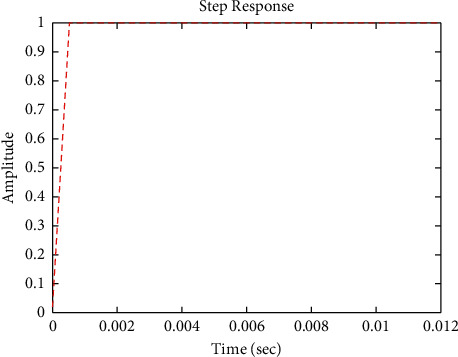
Step response of the closed form for transfer function *G*_2_(*s*).

**Figure 12:**
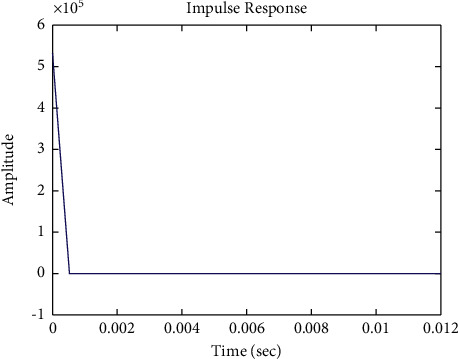
Impulse response of the closed form for transfer function *G*_2_(*s*).

**Figure 13:**
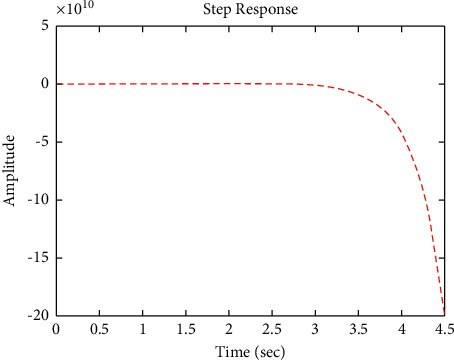
Step response of the open form for transfer function *G*_2_(*s*).

**Figure 14:**
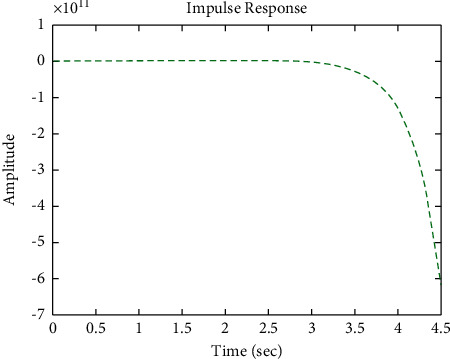
Impulse response of the open form for transfer function *G*_2_(*s*).

Figure 12 shows the impulse response for the closed form (the signal is highlighted in blue) is better than the open form (the signal is highlighted in green in Figure 14). The value of the amplitude of the closed form in this case is zero, and the closed form reaches a state of the steady. For the PID controller, the closed form responds well. The value of the amplitude of the open form in this case is large, and the open form does not reach a state of the steady.  Figure 11 shows the step response for the closed form (the signal is highlighted in red) is better than that for the open form (the signal is highlighted in red in Figure 13). The value of amplitude of the closed form in this case is 1, and the closed form reaches a state of the steady. For the PID controller, the closed form responds well. Meanwhile, the open form cannot respond well.

**Figure 16:**
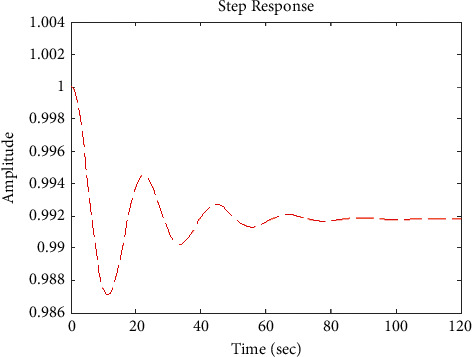
Step response of the closed form for transfer function *G*_3_(*s*).

**Figure 17:**
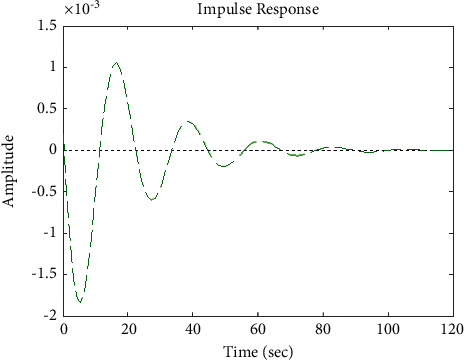
Impulse response of the closed form for transfer function *G*_3_(*s*).

**Figure 18:**
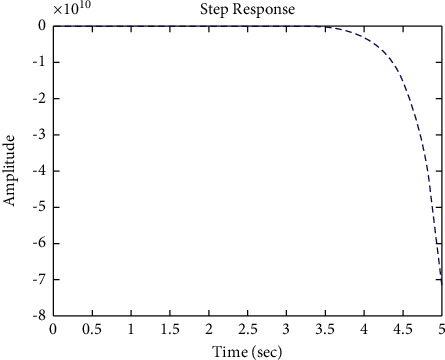
Step response of the open form for transfer function *G*_3_(*s*).

**Figure 19:**
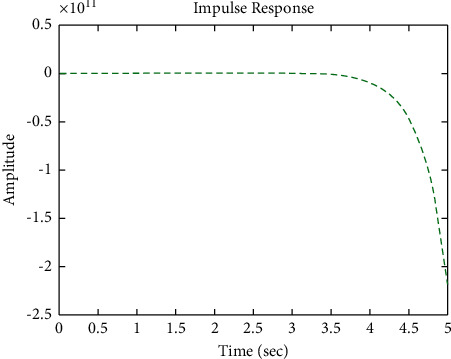
Impulse response of the open form for transfer function *G*_3_(*s*).

Figure 17 shows the impulse response for the closed system (the signal is highlighted in green) is better than the open form (the signal is highlighted in green in Figure 19). The value of the closed form in this case is zero, and the closed form reaches a state of the steady. For the PID controller, the closed form responds well. The value of the amplitude of the open form in this case is large, and the open form does not reach a state of the steady.  Figure 16 shows the step response for the closed form (the signal is highlighted in red) is better than that for the open form (the signal is highlighted in blue in Figure 18). The value of the amplitude of the closed form in this case is 0.992, and the closed form reaches a state of the steady. For the PID controller, the closed form responds well. Meanwhile, the open form cannot respond well.

**Figure 21:**
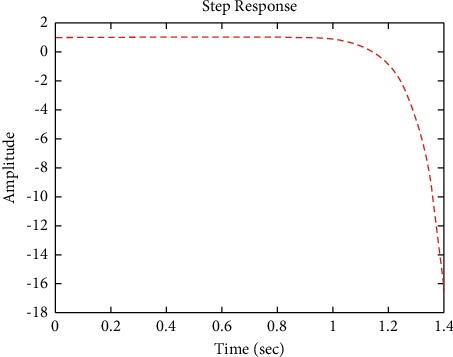
Step response of the closed form for transfer function *G*_4_(*s*).

**Figure 22:**
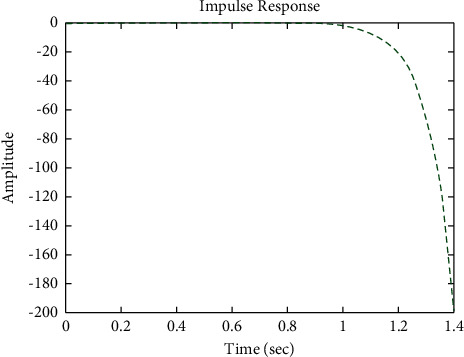
Impulse response of the closed form for transfer function *G*_4_(*s*).

**Figure 23:**
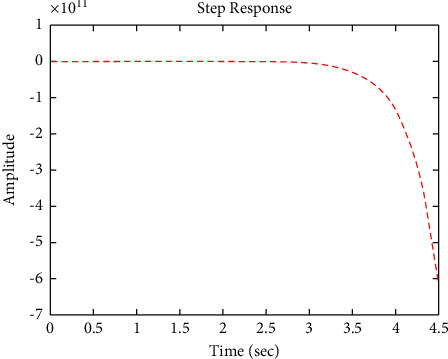
Step response of the open form for transfer function *G*_4_(*s*).

**Figure 24:**
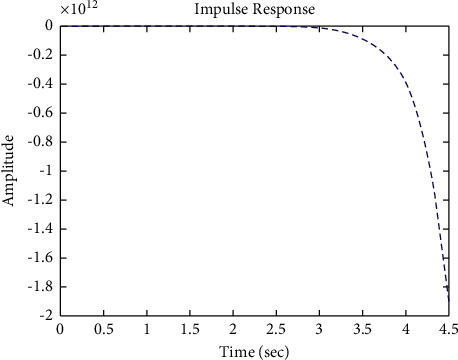
Impulse response of the open form for transfer function *G*_4_(*s*).

Figure 22 shows the impulse response for the closed form (the signal is highlighted in green) is better than the open form (the signal is highlighted in blue in Figure 24). The value of the amplitude of the closed form in this case is large, and the closed form does not reach a state of the steady. For the PID controller, the closed form does not respond well. The value of the amplitude of the open form in this case is large, and the open form does not reach a state of the steady.  Figure 21 shows the step response for the closed form (the signal is highlighted in red) is better than that for the open form (the signal is highlighted in red in Figure 23). The value of the amplitude of the closed form in this case is large, and the closed form does not reach a state of the steady. For the PID controller, the closed form does not respond well. Meanwhile, the open form cannot respond well.

### 6.2. Part 2: Model Using Neural Networks Application for the Data of PID Controller

The neural network used in this case is based on the data collected from simulation results of the PID controller.

Figures 25–32 show that neural networks application for the data of the PID controller is the best choice for training the network according to a given control method. Results show that values of control methods are relatively consistent with each other, and results have achieved desired requirements.

## 7. Conclusions

To simplify and speed up the design process of control systems in general and Acrobot in particular, the author's choices for the control presented above are consistent with the strategies laid out. Simulinks for the design of closed forms are according to the design process. The analysis for the performance of the system was tested with the PID controller, neural networks application. The purpose of this study is for research and educational purposes. The analysis obtained from curves of 2 responses showed that the strategies for controlling a system were implemented on the simulation software carefully. Neural networks application can be a priority to be selected among above methods. Neural networks application has achieved satisfactory results in training the data of any controller, specifically here the PID controller. It has very good security according to plans outlined. In the process of implementing this topic, simulation results are stable for the system to be considered by the author. In the future, this system can be controlled by more modern algorithms such as the new modified multitracker optimization algorithm to suit more stringent requirements.

## Figures and Tables

**Figure 5:**
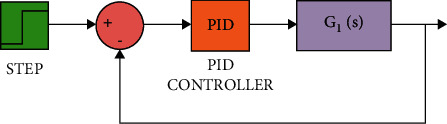
Simulink of the PID controller for transfer function *G*_1_(*s*).

**Figure 10:**
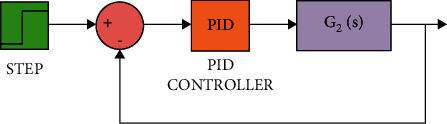
Simulink of the PID controller for transfer function *G*_2_(*s*).

**Figure 15:**
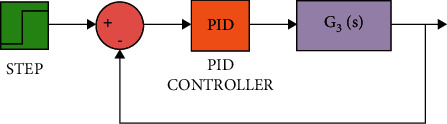
Simulink of the PID controller for transfer function *G*_3_(*s*).

**Figure 20:**
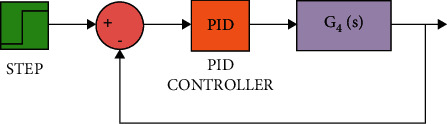
Simulink of the PID controller for transfer function *G*_4_(*s*).

**Figure 25:**
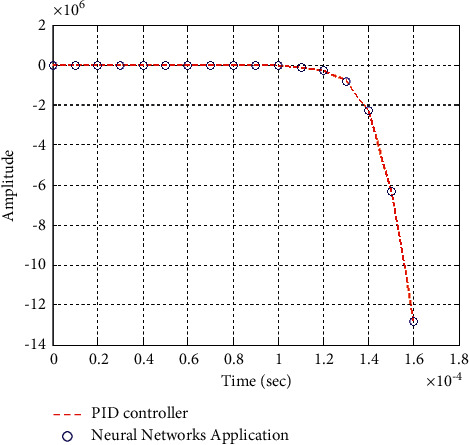
Step response of the closed form “*G*_1_(*s*)”.

**Figure 26:**
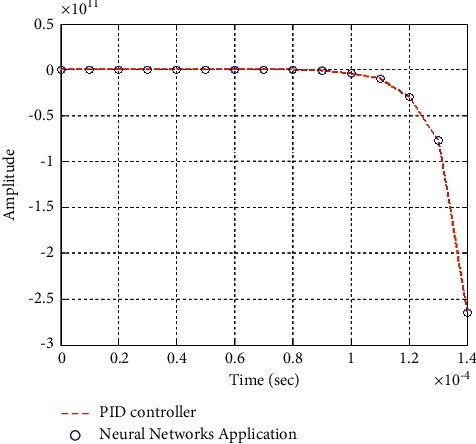
Impulse response of the closed form “*G*_1_(*s*)”.

**Figure 27:**
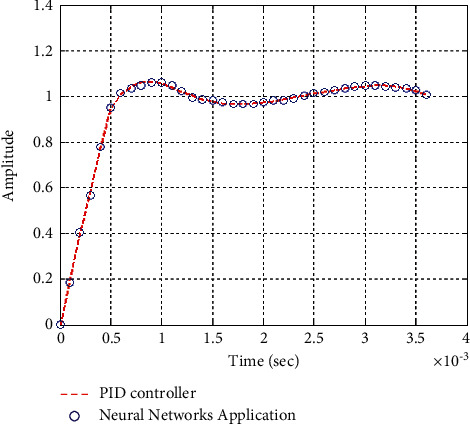
Step response of the closed form “*G*_2_(*s*)”.

**Figure 28:**
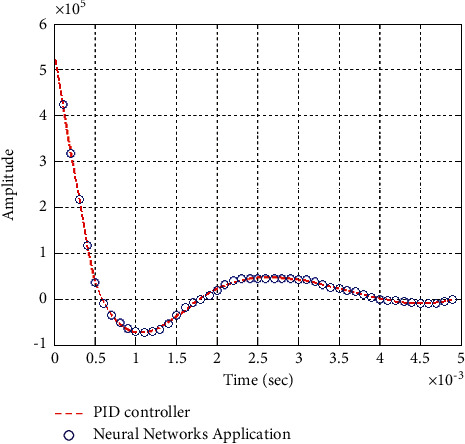
Impulse response of the closed form “*G*_2_(*s*)”.

**Figure 29:**
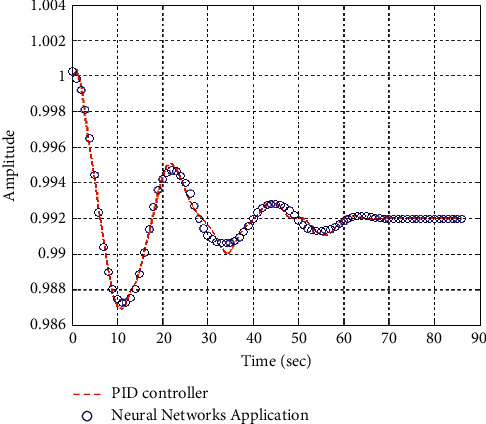
Step response of the closed form “*G*_3_(*s*)”.

**Figure 30:**
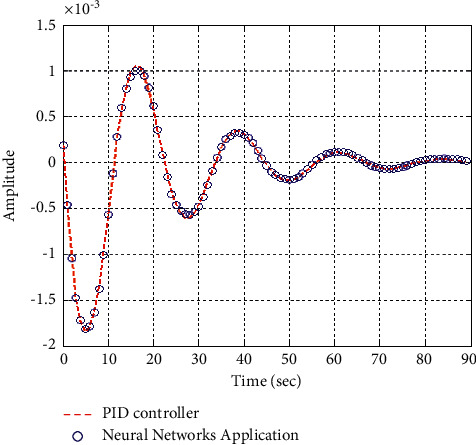
Impulse response of the closed form “*G*_3_(*s*)”.

**Figure 31:**
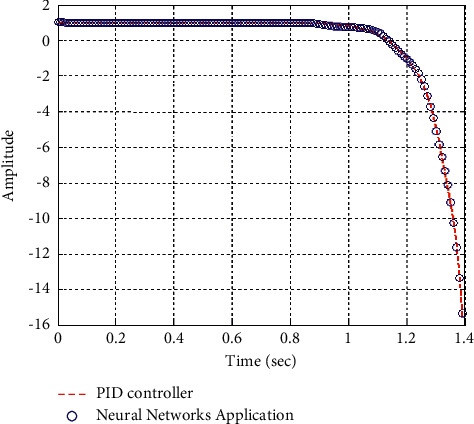
Step response of the closed form “*G*_4_(*s*)”.

**Figure 32:**
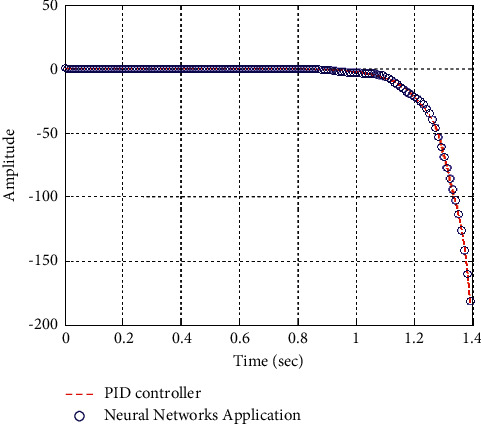
Impulse response of the closed form “*G*_4_(*s*)”.

**Table 1:** Parameters of Acrobot.

Parameters	Values
*m*_1_	0.8 kg
*O*_1_	0.18 m
*O*_*C*1_	0.11 m
*m*_2_	0.2 kg
*O*_2_	0.18 m
*O*_*c*2_	0.09 m
*J*_1_	0.0022 kg·m^2^
*J*_2_	0.00054 kg·m^2^

**Table 2:** Parameters of the model.

Parameters and variables	Described
*q*_1_	Angle of link 1
*q*_2_	Angle of link 2
${\dot{q}}_{1}$	Angle velocity of link 1
${\dot{q}}_{2}$	Angle velocity of link 2
*m*_1_	Mass of link 1
*O*_1_	Length of link 1
*O*_*c*1_	Distance from passive joint to the center of mass of the link 1
*m*_2_	Mass of link 2
*O*_2_	Length of link 2
*O*_*c*2_	Distance from active joint to the center of mass of the link 1
*J*_1_	Moment of inertia link 1
*J*_2_	Moment of inertia link 2
*g*	Gravitational acceleration
*τ*_2_	Torque applied to active joint

## Data Availability

The data used to support the findings of this study are cited as references within the article.
